# Ultrasound-based airway assessment in obese patients as a valuable tool for predicting difficult airway: an observational study

**DOI:** 10.1016/j.bjane.2024.844539

**Published:** 2024-07-10

**Authors:** Ozan Tasdemir, Nazan Kocaoglu, H. Fisun Demir, Fatih Ugun, Ozlem Sagir

**Affiliations:** a Balikesir Atatürk City Hospital, Department of Anesthesiology and Intensive Care Medicine, Altieylul, Balikesir, Türkiye; b Balıkesir University Faculty of Medicine, Department of Anesthesiology and Intensive Care Medicine, Altieylul, Balikesir, Türkiye

**Keywords:** Airway management, Laryngoscopy, Obesity, Ultrasonography

## Abstract

**Background:**

Difficult airway, characterized by difficult mask ventilation and intubation, is common in obese patients undergoing surgery. The purpose of this study was to evaluate and compare the prognostic efficiency of ultrasound-measured anterior cervical soft tissue parameters as an indicator of difficult airway during anesthesia induction in obese patients.

**Methods:**

This prospective, double-blind, observational study was conducted at Balikesir University Faculty of Medicine Hospital between March 2020 and March 2022. A total of 157 patients age ≥ 18 (BMI ≥ 30 kg.m^-2^), without previous head and neck surgery were included in the study. Anterior cervical soft tissue measurements were performed at three levels; minimum distance between the hyoid bone and skin at the level of the hyoid bone; (DSHB), distance between the midpoint of the epiglottis and skin at the level of the thyrohyoid membrane; (DSE), distance between the anterior commissure of vocal cords and skin at the vocal cord level; (DSV). The Han scale was used to assess difficult mask ventilation and the Cormack-Lehane scale was used to assess difficult laryngoscopy.

**Results:**

In the difficult laryngoscopy group, the mean values of DSHB, DSE and DSV were 18.5 ± 3.5, 18.3 ± 3.8, and 18.6 ± 3.4, respectively. The AUC values for DSHB, DSE, and DSV were 0.845, 0.827, and 0.850, respectively. Anterior cervical measurements showed a better predictive value for difficult laryngoscopy compared to difficult mask ventilation.

**Conclusion:**

Ultrasonographic measurements were predictive for difficult laryngoscopy and ventilation with better correlation in laryngoscopy.

## Introduction

Obesity is a global public health concern in modern societies. In elective and surgical procedures, obese patients are frequently encountered.^1^ Obesity may cause various anatomical and physiological changes such as increased adipose tissue deposition in the pharyngeal and palatal soft tissues, decreased pulmonary compliance, increased airway resistance and inadequate ventilation and perfusion.^2^ A difficult airway, which is characterized by difficult mask ventilation and/or difficult intubation, is particularly common. Obesity has been shown to cause a threefold increase in the risk of difficult intubation.^3^ It may lead to rapid oxygen desaturation during airway management, mask ventilation, or after extubation. Anesthetic complications related to difficult laryngoscopy and difficult mask ventilation can result in severe morbidity and mortality and may cause surgical procedure failure and increased risk of an unsuccessful outcome. Early detection of obesity-related difficult airway management may provide time for preparation by the anesthetic and surgical team. Alternative airway management strategies may be planned, appropriate equipment prepared, patient safety increased, and reduction in airway complications may improve surgical outcomes and positively influence patient recovery.

Several studies have shown that the sensitivity of traditional preoperative screening tests for predicting difficult airways is limited.^3^^,^^4^ Ultrasound (US) is a fast, simple, reliable, and cost-effective bedside method. It is widely used for lung and cardiac evaluation, nerve blocks, and central venous catheter insertion in current anesthesia and intensive care practices. During airway assessment, important anatomical structures such as the hyoid bone, thyroid cartilage, cricoid cartilage, and trachea can be evaluated.^5^ In addition, the position and movement of the epiglottis and vocal cords can be assessed. This evaluation can identify landmarks for invasive airway techniques, such as cricothyroidotomy or tracheostomy. Furthermore, it is also possible to measure the distance of various anatomical structures (epiglottis, hyoid bone, and vocal cords) from the skin and the volume of the tongue, which can be useful in predicting difficult airways. However, studies have shown that the sensitivity and specificity of anatomical measurements are variable, and no single measurement is more predictive than others.^6^ Furthermore, in obese patients who are at higher risk for airway complications, noninvasive techniques such as ultrasonography may be helpful in determining airway difficulties and may prove to be valuable for clinical practice. However, the predictive role of soft tissue measurements with US in the determination of difficult mask ventilation and difficult laryngoscopy in obese patients has only been investigated in a few studies.

In this regard, the present prospective observational study aimed to evaluate and compare the predictive value of US-measured anterior cervical soft tissue parameters as markers of difficult laryngoscopy and difficult ventilation in obese patients under general anesthesia.

## Methods

### Ethical approval

The ethical approval (Decision n° 2020/16) of this prospective observational, double-blind study was provided by Balikesir University Faculty of Medicine, Clinical Research Ethics Committee Balikesir, Turkey on 05.02.2020 and registered in ClinicalTrials.gov (NCT05896098). All study participants gave informed written consent, and the research was conducted in accordance with the Helsinki Declaration and Good Clinical Practice Guidelines.

### Study design

Patients aged ≥ 18 years, BMI ≥ 30 kg.m^-2^ and American Society of Anesthesiologists (ASA) I–II, who were scheduled for elective surgery under general anaesthesia between March 2020 and March 2022 in our department were included in the study. The study excluded patients who experienced facial and neck trauma, a history of any kind of head and neck surgery and malignancies, tracheotomy, a history of difficult intubation, non-cooperative patients and pregnancy.

### Preoperative evaluation and screening tests

Sex, age, height, weight, ASA and obesity group according to BMI (obese ≥ 30 kg.m^-2^, severely obese ≥ 35 kg.m^-2^, morbidly obese ≥ 40 kg.m^-2^ or > 35 kg.m^-2^ with comorbidity, and superobesity ≥ 50 kg.m^-2^) were recorded. Airway screening tests, including evaluation of thyromental distance, sternomental distance, hyomental distance, and mouth opening were performed. In addition, neck circumference was measured at the level of the cricoid cartilage. Modified Mallampati Score (MMS) and Upper Lip Bite Test (ULBT) were performed. ULBT measurements were classified as Class I if the upper lip completely closed the vermilion when bitten, Class II if the upper lip was bitten below the junction of the upper lip and vermilion, and Class III if the upper lip could not be bitten. Clinical airway screening tests and US measurements were performed by two different anesthetists who were blinded to each other's data.

### Ultrasound measurements

**Figure 1:**
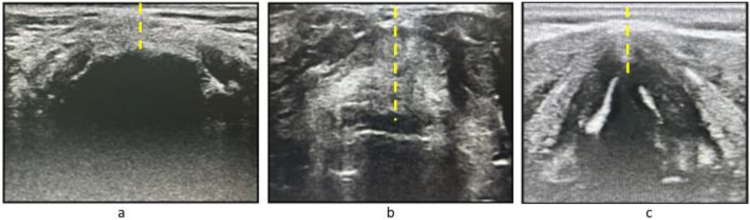
Ultrasound distances; (a) DSHB, minimum Distance from Skin to the Hyoid Bone, (b) DSE, Distance from Skin to the Epiglottis midpoint; (c) DSV, Distance from Skin to the Vocal cord anterior junction.

Anterior cervical soft tissue measurements were performed with a linear 13–6 MHz US probe (Sonosite M-Turbo) at three levels in the craniocaudal axis and in the transverse plane by the same anesthetist with the patient supine on the operating table, with head and the neck in a neutral position. The following soft tissue distances were measured and recorded: minimum Distance between the hyoid bone and the skin at the level of the hyoid bone (DSHB); distance between the midpoint of the epiglottis and the skin (DSE); between the hyoid bone and thyroid cartilage at the level of the thyrohyoid membrane; distance between the anterior commissure of the vocal cords and the skin (DSV) at the vocal cord level (Figure 1).

Prior to the main study, ultrasound measurements were studied and performed on 50 patients by an experienced anesthetist.

### Anesthesia management

After routine fasting (solid food ≥ 8 h, clear liquids ≥ 2 h) all patients were monitored using electrocardiography, noninvasive blood pressure, pulse oximetry, and temperature monitoring on the day of the operation. Train of Four (TOF) was used for neuromuscular monitoring. Preoxygenation was performed for 3 min while the patient was in ramp position. After standard anesthesia induction (propofol 2 mg.kg^-1^, fentanyl 2 mcg.kg^-1^, rocuronium bromide 0.6 mg.kg^-1^ intravenously), TOF measurement was initiated immediately, and mask ventilation was continued with a disposable plastic mask until the TOFR reached 0. Han Scale (Class 1: Can be ventilated with mask; Class 2: Can be ventilated with mask and oral airway or other equipment; Class 3: Can be ventilated with the need for a second person for ventilation; Class 4: Cannot be ventilated with mask) was used to evaluate mask ventilation. Han Scale 3–4 was considered as Difficult Mask Ventilation (DMV). Laryngoscopy was performed using an appropriate size Macintosh blade at TOFR = 0. Mask ventilation and laryngoscopy were performed by the same anesthetist with at least 2 years of experience blinded to US measurements. Laryngoscopic view (without any external maneuver) was classified via the Cormack-Lehane (CL) Classification (Class 1: Vocal cords are completely visible Class 2a: Vocal cords are partially visible Class 2b: Arytenoid cartilages or posterior vocal cords are visible; Class 3a: Epiglottis can be removed 3b: Epiglottis adherent to the pharynx or only its tip is visible; Class 4: No glottic structures are seen) and ≥ 2b was considered difficult laryngoscopy.

### Statistical analysis

The power and alpha values were 90% and 5% respectively; and the sample size was 140, with reference to studies^7^^,^^8^ (G-Power program version 3.1.9.7). Frequency tables for categorical variables and descriptive statistics for continuous variables were calculated. Pearson's Chi-Square test was used to analyze categorical data in terms of groups, and the Shapiro-Wilk normality test was used to analyze the distribution of continuous variables. The Mann-Whitney *U* test was used to compare variables between two groups when the normal distribution assumption was not met, and the Kruskal-Wallis test was used if there were more than two groups. Spearman's correlation coefficient (*r*) was calculated to analyze the correlation between US measurements and the DMV and difficult laryngoscopy. The results were scored as zero (0.0–0.1), weak (0.1–0.3), moderate (0.3–0.6), strong (0.6–0.9), and very strong (0.9–1). The predictive value of the US measurements for DMV and difficult laryngoscopy was estimated using the area under the receiver operating characteristic curve (AUC). Optimal cut-off values were determined using Youden's index. The IBM SPSS version 25.0 package program was used in the analyses; *p <* 0.05 was considered statistically significant.

## Results

**Figure 2:**
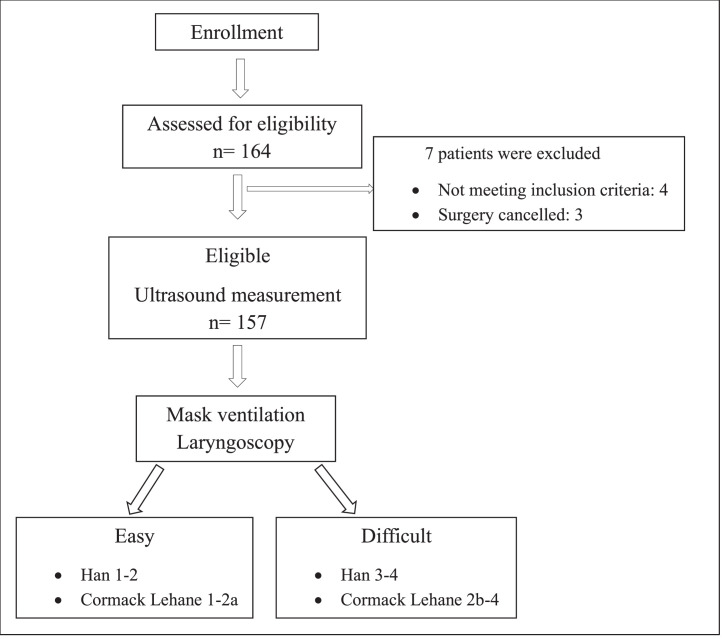
Flowchart of the study.

A total of 157 obese patients who underwent elective surgery were included in this study (Figure 2). The incidence of DMV and difficult laryngoscopy was 16.56% (n = 26) and 32.48% (n = 51), respectively. There was no statistical difference between easy and difficult laryngoscopy groups in terms of age, gender, BMI, ASA physical status, OSAS, surgery type and obesity classification (*p* > 0.05) (Table 1). But there was a statistical difference between the easy and difficult mask ventilation groups in terms of gender and ASA physical status (*p* < 0.05) (Table 1).

**Table 1:** Demographical and preoperative data.

	Han 1‒2	Han 3‒4		CL 1‒2a	CL 2b‒4	
	Group Easy	Group Difficult	*p*	Group Easy	Group Difficult	*p*
	n = 131	n = 36		n = 106	n = 51	
**Age** (years)	39.02 ± 12.99	44.96 ± 14.37	0.038	38.62 ± 13.43	42.88 ± 12.89	0.051
**Gender**			0.024			0.792
Male	28 (21.4%)	11 (42.3%)		27 (25.5%)	12 (23.5%)	
Female	103 (78.6%)	15 (57.7%)		79 (74.5%)	39 (76.5%)	
**Body Mass Index** (kg.m^-2^)	42.47 ± 5.95	42.01 ± 7.33	0.50	42.53 ± 6.03	42.13 ± 6.52	0.406
**ASA physical status**			0.01			0.252
I	98 (74.8%)	13 (50%)		78 (73.59%)	33 (64.70%)	
II	33 (25.2%)	13 (50%)		28 (26.41%)	18 (35.30%)	
**Obstructive Sleep Apnea Syndrome**	3 (2.3%)	1 (3.8%)	0.64	2 (1.88%)	2 (1.96%)	0.596
**Surgery**			0.09			0.240
Bariatric	101 (77.1%)	16 (61.5%)		82 (77.36%)	35 (68.63%)	
Others	30 (22.9%)	10 (38.5%)		24 (22.64%)	16 (31.37%)	
**Classification of Obesity**			0.09			0.389
Obese	26 (19.8%)	9 (34.6%)		22 (20.75%)	13 (25.50%)	
Severe Obese	93 (71%)	15 (57.8%)		74 (69.81%)	34 (66.66%)	
Morbid Obese	10 (7.6%)	1 (3.8%)		9 (8.49%)	2 (3.92%)	
Super Obese	2 (1.5%)	1 (3.8%)		1 (0.95%)	2 (3.92%)	

Values are mean ± SD, numbers or numbers (per cent). CL, Cormack-Lehane; ASA, American Society of Anesthesiologists; *p* < 0.05.

According to the Han Scale, 131 of 157 patients (83.44%) were classified as having easy mask ventilation. The DSHB, DSE, and DSV measurements were significantly longer in the difficult mask ventilation group than in the easy mask ventilation group (*p* < 0.001)*.* In addition, based on the Cormack-Lehane classification, 106 of the 157 patients (67.5%) were classified as having easy laryngoscopy. The DSHB, DSE, and DSV measurements were significantly longer in the difficult laryngoscopic group than in the easy laryngoscopic group (*p* < 0.001) (Table 2).

**Table 2:** Ultrasound distances in difficult mask ventilation and difficult laryngoscopy groups.

	Han 1‒2	Han 3‒4		CL 1‒2a	CL 2b‒4	
	Group Easy	Group Difficult	*p*	Group Easy	Group Difficult	*p*
	n = 131	n = 26		n = 106	n = 51	
**DSHB (mm)**	15.4 ± 3.0	17.7 ± 3.6	0.0016	14.5± 2.0	18.5 ± 3.5	0.0002
**DSE (mm)**	14.9 ± 3.1	18.1 ± 3.2	0.0000	14.0± 1.9	18.3 ± 3.8	0.0003
**DSV (mm)**	15.3 ± 3.0	17.7 ± 3.5	0.0028	14.2± 1.9	18.6 ± 3.4	0.0001

Values are mean ± SD, numbers. CL, Cormack-Lehane; DSHB, Minimum Distance from the Skin to the Hyoid Bone; DSE, Distance from the Skin to the Epiglottis midpoint; DSV, Distance from the Skin to the Vocal cord anterior junction; *p* < 0.05.

A positive and significant correlation was found between ultrasound measurements and both difficult mask ventilation and laryngoscopy (Table 3). The DSHB and DSV were weakly correlated with difficult mask ventilation, whereas the DSE was moderately correlated. Difficult laryngoscopy showed a moderate correlation with DSHB, DSE, and DSV (Table 3).

**Table 3:** The correlations between ultrasound distances and difficult mask ventilation and difficult laryngoscopy.

		Difficult Mask Ventilation	Difficult Laryngoscopy
		n = 26	n = 51
**DSHB**	*r*	0.252	0.560
	*p*	0.001^a^	0.000^a^
**DSE**	*r*	0.339	0.531
	*p*	0.000^a^	0.000^a^
**DSV**	*r*	0.003^a^	0.569
	*p*	0.239	0.000^a^

DSHB, Minimum Distance from the Skin to the Hyoid Bone; DSE, Distance from the Skin to the Epiglottis midpoint; DSV, Distance from the Skin to the Vocal cord anterior junction; n, numbers; Spearman's rho correlation test ^a^*p* < 0.05.

**Figure 3:**
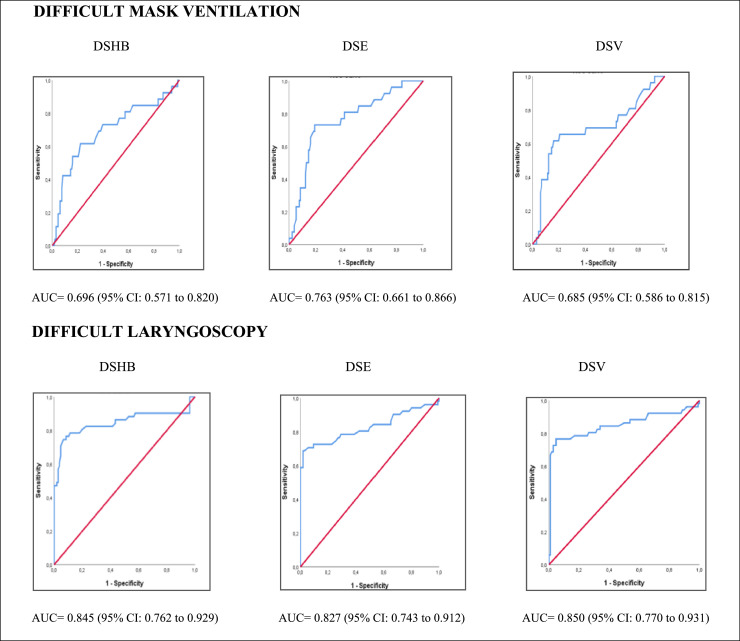
Receiver operating characteristic curve analyzes of the three ultrasound measurements and difficult mask ventilation and difficult laryngoscopy. DSHB, Minimum Distance from Skin to the Hyoid Bone; DSE, Distance from Skin to the Epiglottis midpoint; DSV, Distance from Skin to the Vocal cord anterior junction.

For the difficult mask ventilation group, the cut-off values for DSHB, DSE and DSV were 17.3 mm, 17.1 mm, and 18.3 mm, respectively, as determined by Youden's index. According to the ROC analysis, the AUC values were 0.696, 0.763, and 0.685, respectively. In the difficult laryngoscopy group, the cut-off values for DSHB, DSE and DSV were 16.9 mm, 17.5 mm, and 16.8 mm, respectively, and the corresponding AUC values were 0.845, 0.827, and 0.850. Anterior cervical measurements demonstrated a superior predictive value for difficult laryngoscopy than for difficult mask ventilation (Figure 3).

The MMS and neck circumference were significantly different between the easy and difficult mask ventilation groups (*p* < 0.05). The MMS had a sensitivity of 89.8%, specificity of 30.6%, PPV of 74%, NPV of 57.7%, and AUC of 0.659. Neck circumference measurements had a sensitivity, specificity, PPV, NPV, and AUC of 59.5%, 65.4%, 89.7%, 24.3%, and 0.625, respectively. The cutoff measure was 45.5 cm (*p* < 0.05). The MMS and ULBT significantly differed between the easy and difficult laryngoscopy groups (*p* < 0.001). MMS had a sensitivity of 79.9%, specificity of 59.2%, PPV of 81.1%, NPV of 56.9% and AUC of 0.690; ULBT had a sensitivity of 71.1%, specificity of 100%, PPV of 100%, NPV of 15.7%, and AUC of 0.578 (*p* < 0.05). There were no significant differences between difficult mask ventilation and difficult laryngoscopy in thyromental, hyomental, sternomental distance and mouth opening measurements on clinical airway screening tests (*p* > 0.05).

## Discussion

This prospective, observational study assessed the predictive value of ultrasound measurements for difficult mask ventilation and laryngoscopy in obese patients. The prevalence of difficult mask ventilation and laryngoscopy was 16.56% and 32.48%, respectively. Ultrasound measurements of the anterior cervical soft tissues at the level of the hyoid bone, thyrohyoid membrane and vocal cords were predictive of difficult mask ventilation and difficult laryngoscopy with better correlation for laryngoscopy.

The literature reports that the incidence of difficult laryngoscopy varies from 9.5–16.7% in the general population and 12–31% in obese patients.^8^, ^9^, ^10^, ^11^ In our study the incidence of difficult laryngoscopy in obese patients was 32.48%, which is above the range reported in the literature. The incidence of difficult laryngoscopy may differ depending on factors such as head positioning, lack of neuromuscular blockade monitoring, patient characteristics, practitioner experience, and the use of maneuver during direct laryngoscopy (including the Sellick maneuver).^12^ These varying factors may contribute to different results in the occurrence of difficult laryngoscopy. Komatsu et al performed laryngoscopy according to the Cormack-Lehane classification without external laryngeal compression and found that the incidence of difficult laryngoscopy (CL III–IV) was 31%.^8^ Wang et al. reported a 28.85% incidence rate of difficult laryngoscopy (CL ≥ 2b).^8^^,^^13^ External compression was not applied during laryngoscopy image recording, and a CL ≥ 2b was classified as difficult laryngoscopy. Reports also have shown that the incidence of Cormack-Lehane III–IV increases in correlation with BMI, determining a ratio of 20.4% in obese and 44.4% in super-morbid obese patients.^14^ The mean BMI was > 40 kg.m^-2^ in all our patients, which may have contributed to the higher incidence of difficult laryngoscopy.

Previous studies evaluating the relationship between ultrasound soft tissue measurements of the anterior neck and difficult laryngoscopy in obese and non-obese patient groups have shown different results.^2^^,^^15^, ^16^, ^17^, ^18^ Wu et al showed a strong correlation between difficult laryngoscopy and US measurements at the level of the hyoid bone, thyrohyoid membrane and vocal cords in non-obese Chinese patients with the head in neutral position.^15^ In a different group of non-obese patients, DSE with the head in the sniffing position was identified as the best predictor.^16^ However, Ezri et al. showed that DSV was the best predictor of difficult laryngoscopy in patients with BMI > 35 kg.m^-2^.^17^

In another study, the DSE was found to be a predictive measure of difficult laryngoscopy in obese patients.^18^ DSV was not significant between easy and difficult laryngoscopy groups in obese patients in the American population.^8^ In the present study, the AUC values of DSHB, DSE and DSV were above 0.8. These findings suggested that the measurement of anterior cervical soft tissue using ultrasound at three different levels may be helpful in difficult laryngoscopy. However, given the similarity in the ROC analysis results, we conclude that no single-level measurement was superior to the others. In the current study, the cut-off values of DSHB, DSE, and DSV for difficult laryngoscopy were 16.9, 17.5 and 16.8 mm, respectively. Two studies in South American and Thai populations have shown that the cutoff value for DSE in obese patients with a BMI greater than 35 kg.m^-2^ is 29.3 mm and 13 mm, respectively.^2^^,^^18^ Anthropometric measurements have identified differences in body structure and craniofacial features among racial groups. Furthermore, there are ethnic differences in the morphology of maxillary and mandibular bones. Additionally, sleep studies have demonstrated different craniofacial features in different ethnic groups predicting upper airway collapse. In addition to ethnic differences, the skill and experience of the anesthetist and the methodology of the US assessments may have influenced the results of these studies.^19^

The literature reports an incidence of difficult mask ventilation of 3–15% in the general population and 8.8–14% in obese patients.^20^, ^21^, ^22^, ^23^ In this study, the incidence of difficult mask ventilation was 16.56%. Our study included mainly morbid obese patients which may have contributed to this increase. Few studies have examined the link between DMV and ulltrasound in the general population. Alessandri et al have shown that the predictive value of DSHB (AUC = 0.608) is better than other measurements in the general population.^24^ Bianchini et al assessed the difficulty of mask ventilation before and after using neuromuscular blocking agents.^25^ The same study, which examined 12 different US measurements, showed that tongue thickness was more predictive in non-curarized patients (head hyperextension) and the hyomental distance was more predictive in curarized patients (active mandibular subluxation). However, when BMI was included in the multivariate analysis, US measurements at the hyoid bone level showed no predictive value. Based on our findings, DSHB and DSV showed a poor correlation with difficult mask ventilation, whereas DSE showed a moderate correlation. In the ROC analysis, the DSE (AUC = 0.763) had a better predictive value for difficult mask ventilation.

Previous studies have shown that neck circumference is predictive of difficult mask ventilation. Leoni et al reported that neck circumference > 46 cm in patients with a BMI > 30 kg.m^-2^ was a predictor of difficult mask ventilation.^21^ In another study, neck circumference > 43 cm was found to be predictive of difficult mask ventilation in obese patients.^22^ In the current study, the cutoff value for neck circumference was 45.5 cm and the AUC was 0.625. According to our results, neck circumference measurements should be evaluated together with other airway screening tests in obesity.

The Modified Mallampati Score (MMS) is frequently used by anesthetists to diagnose difficult airways owing to its easy and simple administration. However, for accurate assessment, patients must sit and open their mouths as widely as possible without making any sounds.^26^ A meta-analysis concluded that the predictive value of the MMS is an inadequate screening test for predicting difficult airway or difficult laryngoscopy and should be evaluated in association with other methods.^27^ Another study investigating the relationship between direct laryngoscopy and BMI, suggested the need for MMS to assess difficult intubation by highlighting its agreement with the CL classification.^28^ In the current study, the ROC analyses of MMS for difficult mask ventilation and difficult laryngoscopy were AUC 0.659 and 0.690 respectively.

The upper lip bite test is a simple bedside test that does not require positional or vocal restrictions, special equipment or additional lighting.^29^ Khan et al showed that the ULBT had higher specificity and accuracy than the MMS.^30^ Similarly, a study involving morbidly obese patients showed comparable results.^1^ In the present study, the AUC value of the ULBT for difficult laryngoscopy was 0.578. Based on these results, we recommend MMS and ULBT in combination with other screening tests for the evaluation of difficult airways for obesity.

### Limitations

Our study has several limitations. First, the anesthetist performing the US measurements was not officially trained by a radiologist. Second, although there was a significant correlation between difficult intubation, difficult laryngoscopy and CL classification, we could have used a scoring system to standardize other conditions affecting difficult intubation. Furthermore, the number of female patients was approximately threefold higher than of male patients. Statistical comparison among same gender patients was not conducted. Finally, the study was performed in a single center and only in the Turkish population.

## Conclusion

This study assessed the relationship between difficult mask ventilation and difficult laryngoscopy by measuring anterior neck soft tissue thickness at the level of the hyoid bone, thyrohyoid membrane and vocal cords using ultrasound in patients with a BMI ≥ 30 kg.m^-2^. Among the measured parameters, DSHB, DSE, and DSV were predictive for difficult laryngoscopy and ventilation with better correlation in laryngoscopy.

Increased ultrasound usage is recommended in daily anesthetic practice to provide a safe airway in obese patients. In the future, protocols for the use of this method can be established by international, multicenter studies involving different investigators and different ethnic populations.

## Conflicts of interest

The authors declare no have conflicts of interest.
